# Efficacy of alginate-based reflux suppressant and magnesium-aluminium antacid gel for treatment of heartburn in pregnancy: a randomized double-blind controlled trial

**DOI:** 10.1038/srep44830

**Published:** 2017-03-20

**Authors:** Pontip Meteerattanapipat, Vorapong Phupong

**Affiliations:** 1 Department of Obstetrics and Gynecology, Faculty of Medicine, Chulalongkorn University, Rama IV Road, Pathumwan, Bangkok 10330, Thailand

## Abstract

The aim of this study was to compare the therapeutic efficacy of alginate-based reflux suppressant and magnesium-aluminium antacid gel for treatment of heartburn in pregnancy. A double-blinded, randomized, controlled trial was conducted. One hundred pregnant women at less than 36 weeks gestation with heartburn at least twice per week were randomized to either alginate-based reflux suppressant or to magnesium-aluminium antacid gel. Details of heartburn were recorded before beginning the treatment and the second week of study. Primary outcome measure was the improvement of heartburn frequency after treatment and secondary outcome were the improvement of heartburn intensity, quality of life, maternal satisfaction, maternal side effects, pregnancy and neonatal outcomes. There was no difference between treatment and control groups in improvement of heartburn frequency (80% vs 88%, p = 0.275), 50% reduction of frequency of heartburn (56% vs 52%, p = 0.688), improvement of heartburn intensity (92% vs 92%, p = 1.000) and 50% reduction of heartburn intensity (68% vs 80% cases, p = 0.075). There were also no significant differences in quality of life, maternal satisfaction, maternal side effects, pregnancy and neonatal outcomes. Alginate-based reflux suppressant was not different from magnesium-aluminium antacid gel in the treatment of heartburn in pregnancy.

Heartburn or pyrosis is a burning or painful sensation in the upper part of the digestive tract including the throat^1^,^2^,^3^. It is one of the most common gastrointestinal symptoms in pregnant women. The symptoms of heartburn in pregnancy may be frequent, severe and distressing, but serious complications are rare^3^. The worldwide incidence of heartburn in pregnancy is 17% to 80%^2^ and it can occur in all trimesters of pregnancy^2^. The pathogenesis of heartburn in pregnancy involves decreasing lower oesophageal sphincter pressure. The increased circulating progesterone during pregnancy causes lower oesophageal sphincter relaxation^4^,^5^. In addition, the enlarging gravid uterus causes increased intra-abdominal pressure. The normal compensatory response of the lower oesophageal sphincter to accommodate this change is impaired during pregnancy^6^. Abnormal gastric emptying or delayed small bowel transit might also contribute to heartburn in pregnancy^1^. Diagnosis is usually made on symptoms alone, women with more severe illness may undergo diagnostic tests such as upper gastro-intestinal endoscopy and the condition usually resolves after delivery^3^.

Many interventions have been used for the treatment of heartburn in pregnancy. These interventions include changes in diet, lifestyle modification and medications. Common drugs used for the treatment of heartburn in pregnancy include antacids, sucralfate, histamine 2-receptor antagonists, promotility drugs, proton pump inhibitors, and a raft-forming alginate reflux suppressant. However, there has been no evidence-based recommendation for the treatment of heartburn in pregnancy^2^. The Cochrane review in 2015, which includes nine small studies (which involved data from only four small studies), indicates that there are limited data suggesting that heartburn in pregnancy could be completely relieved by pharmaceutical treatment^2^. However, antacids are considered as the first-line medication^1^,^7^. Antacids have no teratogenic effect in animal studies and therefore considered safe to use in pregnancy^8^.

Alginate-based reflux suppressants are licensed for use in pregnant women to combat the frequent symptoms of heartburn and regurgitation. It is equal to or significantly better than traditional antacids for relieving heartburn symptoms^7^. Alginate-based reflux suppressants are designed to provide symptom relief by forming a physical barrier on top of the stomach contents in the form of a neutral floating gel or raft. The advantage of alginate-based reflux suppressants over antacids alone is that they provide rapid and longer lasting symptom relief 9. Due to the physical mode of action and long-term experience, these products are shown to be safe to use in pregnancy^10^. There has been one multicenter, prospective, open-label, and baseline-controlled study of liquid alginate-based reflux suppressants in the treatment of heartburn in pregnant women. The efficacy of the study medication was rated by the investigator (primary endpoint) and patient. Treatment was deemed to be a success in 91% of patients as judged by the investigator and 90% when assessed by the patient themselves^11^.

To date, there has been no randomized controlled trial to compare the efficacy of alginate-based reflux suppressants to magnesium-aluminium antacid gel for treatment of heartburn in pregnancy. Thus, the primary aim of this study was to compare the reduction of heartburn frequency after treatment. Secondary aims were to compare the reduction of heartburn intensity, quality of life, maternal satisfaction, maternal side effects, pregnancy and neonatal outcomes.

## Subjects and Methods

This study was a randomized, double blinded, controlled trial conducted at the Department of Obstetrics and Gynecology, Faculty of Medicine, Chulalongkorn University, Bangkok, Thailand, between June 1, 2015 and July 31, 2016. This study was approved by the Research Ethics Committee of the Faculty of Medicine, Chulalongkorn University. The methods were performed in accordance with approved guidelines. Written informed consent was obtained from all participants. This clinical trial was registered at ClinicalTrials.gov (Clinical trials registration: NCT02470117 date June 5, 2015).

Pregnant women aged 18 to 40 years who presented to antenatal care clinic in our hospital with the diagnosis of heartburn and less than 36 weeks of gestation were invited to join this study. Pregnant women with medical diseases, with study drug contraindications and allergies to alginate-based reflux suppressant and magnesium-aluminium antacid gel were excluded. Participants were advised to undergo lifestyle modification including reducing risk factors of heartburn symptoms such as tobacco and alcohol abstinence, avoiding postprandial recumbent and trigger foods ex. spicy foods, citrus products, fatty or fried foods, carbonated beverages, coffee or other caffeinated beverages. At 1-week follow-up, participants who had persisting or worsened symptoms (heartburn frequency at least two times per week) were included into the study.

After the study was approved, eligible women who gave informed consent were enrolled. Pregnant women were randomized into two groups: treatment or control group. A randomization scheme was generated by random number table using a block-of-four technique. The co-investigator, who had no contact with patients, generated the allocation sequence prior to the study. The primary investigator enrolled and assigned participants to their respective groups. Alginate-based reflux suppressant and magnesium-aluminium antacid gel were prepared prior to the study by a pharmacist who had no involvement in the study. 10 ml of alginate-based reflux suppressant (Liquid Gaviscon^®^ Reckitt Benckiser Healthcare (UK) Ltd, Hull, UK) contains 500 mg sodium alginate, 267 mg sodium bicarbonate and 160 mg calcium carbonate and 5 ml of magnesium-aluminium antacid gel (Maalox ^®^ Olic (Thailand) Co., Ltd., Thailand) contains 120 mg magnesium hydroxide and 220 mg aluminium hydroxide. Each 840 ml of alginate-based reflux suppressant and 840 ml of magnesium-aluminium antacid gel was put into 2 opaque bottles. The bottles were put into an opaque bag. As soon as a study subject met the inclusion criteria, the primary investigator proceeded to select a sequentially numbered opaque bag.

Opaque bags containing 2 bottles of alginate-based reflux suppressant or magnesium-aluminium antacid gel (identical in size, shape and color) were sequentially labeled and used for participants with 2-week follow-up. To ensure randomization, each bag was distributed in sequential numerical order. Both health care providers and study participants were masked to treatment assignment. Alginate-based reflux suppressant was assigned to the treatment group and magnesium-aluminium antacid gel to the control group. Drug dose was 15 ml oral three times after meal and before bedtime. Treatment was continued for 2 weeks. Treatment assignment was not revealed until data collection was completed. Participants recorded details of heartburn (frequency and intensity) and side effects in a diary chart. Participants were asked to return the bottles and diary chart at the end of study to evaluate compliance. Good compliance was defined as participants who completed the required medication doses. Pregnancy and neonatal outcomes were followed-up until delivery.

The primary outcome was to assess the reduction of heartburn frequency after treatment. Secondary outcomes were to assess the reduction of heartburn intensity measured by 100-mm visual analogue scale after treatment, health-related quality of life was self-evaluated using the Optum™ SF-12v2^®^ Health Survey (Thai version), maternal satisfaction, maternal side effects, pregnancy and neonatal outcomes. Satisfaction answer choices consisted of the following: very satisfactory, satisfactory, neutral, unsatisfactory, and very unsatisfactory. Neonatal outcomes included birth weight, Apgar scores, neonatal intensive care unit (NICU) admission and congenital abnormality.

Sample size calculations were based upon the efficacy from previous studies^8^,^11^. The efficacy of antacid was 69% and the efficacy of alginate-based reflux suppressant was 91%. With adjustments for a withdrawal rate of 10%, a minimum of 50 women in each group were required to detect statistical difference (α = 0.05, β = 0.2). Thus, total 100 women were required in this study.

### Statistical analysis

SPSS version 22 (SPSS Inc, Chicago, IL, USA) was used for statistical analysis. Chi-square test and Fisher-exact test for categorical variables, independent t-test for continuous variables, and Mann-Whitney U test for nonparametric variables were used when appropriate. A p < 0.05 was considered statistically significant. Analysis of the trial was conducted in intent-to-treat (ITT) analysis.

## Results

**Figure 1:**
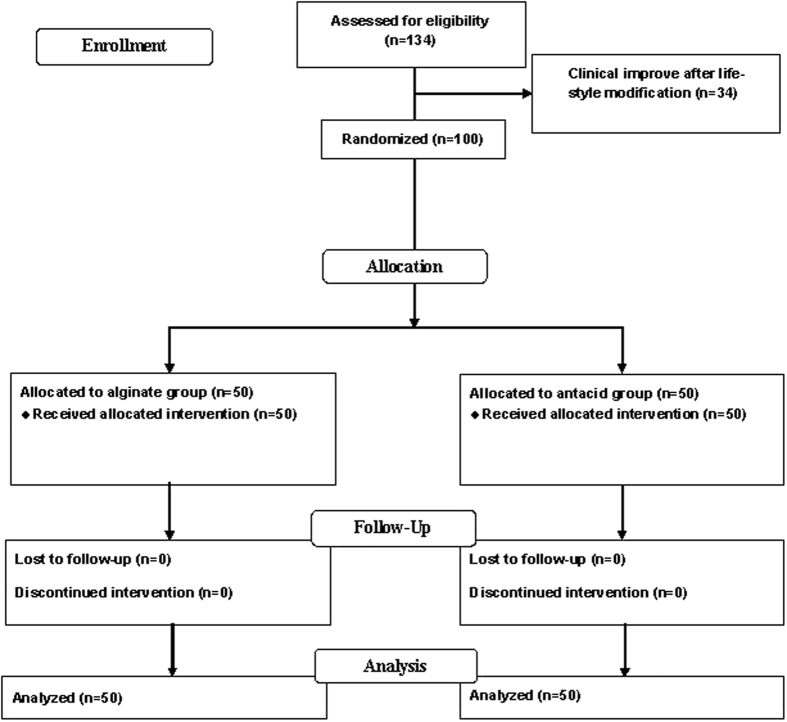
Patient follow-up profile after randomization to either treatment or control group.

One hundred and thirty-four women enrolled in the study (Fig. 1). Thirty-four women improved heartburn symptoms from lifestyle modification after one week. One hundred women who had persistent heartburn symptoms were included into and completed the study. Fifty women were assigned to the alginate-based reflux suppressant group and 50 women were assigned to the magnesium-aluminium antacid gel group. For background characteristics, there were no significant differences between the groups with respect to maternal age, gestational age when heartburn present, pre-pregnancy body mass index (BMI), parity, twin pregnancy, and history of heartburn/dyspepsia before pregnancy (Table 1).

Median numbers of heartburn before treatment were 13 and 12 times per week in the treatment and control groups, respectively (p = 0.649). Median pain score of heartburn before treatment, which was evaluated by 100-mm visual analogue scale, were 42.5 and 43.5 in the treatment and control groups, respectively (p = 0.942) (Table 1).

Table 2 shows the efficacy of treatment, quality of life, maternal satisfaction and side effects between groups. There was no difference between treatment and control groups in improvement of heartburn frequency (80% vs 88%, p = 0.275), 50% reduction of frequency of heartburn (56% vs 52%, p = 0.688), improvement of heartburn intensity (92% vs 92%, p = 1.000) and 50% reduction of heartburn intensity (68% vs 80% cases, p = 0.075) (Table 2). Also, scores for the improvement of quality of life did not differ between treatment and control groups [median change of PCS 7.7 vs 7.6 (p = 0.82), median change of MCS 11.4 vs 6.8, (p = 0.352)] (Table 2).

The satisfactory rate of treatment and side effects were not statistically different between groups (80% vs. 80%, p = 1.000) and (48% vs 50%, p = 0.841), respectively. The two most common side effects in both study groups were constipation (20% and 26%, respectively) and chalk-like taste (22% and 14%, respectively). Pregnancy and neonatal outcome measures included birth weight, APGAR scores and NICU admission did not differ between groups (Table 3). No congenital abnormalities were found in both groups.

## Discussion

This randomized, double blinded, controlled trial compared the therapeutic efficacy of alginate-based reflux suppressant and magnesium-aluminium antacid gel for treatment of heartburn in pregnancy. This study showed no difference in the improvement of heartburn frequency between alginate-based reflux suppressant and magnesium-aluminium antacid gel groups. Similar results were found in terms of the improvement of heartburn intensity, quality of life, maternal satisfaction, maternal side effects and neonatal outcomes.

The improvement of heartburn frequency in this study was 80% in alginate-based reflux suppressant group. This result was similar to previous studies^11^,^12^. Strugala *et al*. performed a multicentre, prospective, open-label, and baseline-controlled study of Liquid Gaviscon in the treatment of heartburn in pregnant women^11^. Treatment was deemed successful in 91% of patients as judged by the investigator and 90% when assessed by the patient themselves. Lindow *et al*. performed an open-label, multicentre study to assess the safety and efficacy of a novel reflux suppressant (Gaviscon Advance) in the treatment of heartburn during pregnancy^12^. Investigator and women efficacy ratings were ′very good′ or ′good′ in 88% and 90% of women.

Quality of life after treatment was improved but not statistically different between both groups. This might be due to the treatment efficacy of both drugs. Overall, the satisfaction of treatment was high (80%) in both groups. This might be due to the minor side effects of drugs.

No neonatal complications occurred in both alginate-based reflux suppressant and magnesium-aluminium antacid gel groups. This confirmed the safety of alginate-based reflux suppressant use in pregnancy as reported from previous studies^11^,^12^. Alginate-based reflux suppressants are licensed for use by pregnant women to combat the frequent symptoms of heartburn and regurgitation. Due to the physical mode of action and long-term experience, this drug is shown to be safe to use in the high risk pregnancy and lactation population^10^,^11^,^13^.

The strength of this study was that it is the first randomized, double blinded, controlled trial conducted to evaluate the efficacy of alginate-based reflux suppressant and magnesium-aluminium antacid gel for treatment of heartburn in pregnancy. Another strength was that we were able to assess patient quality of life before and after treatment, which has yet to be assessed in any current or previous studies. The limitations of this study were that it was a short period of treatment (2-week) and it was underpowered to evaluate some secondary outcomes.

In conclusion, there was no difference between the efficacy of alginate-based reflux suppressant and magnesium-aluminium antacid gel in the treatment of heartburn in pregnancy.

## Additional Information

**How to cite this article**: Meteerattanapipat, P. and Phupong, V. Efficacy of alginate-based reflux suppressant and magnesium-aluminium antacid gel for treatment of heartburn in pregnancy: a randomized double-blind controlled trial. *Sci. Rep.* **7**, 44830; doi: 10.1038/srep44830 (2017).

**Publisher's note:** Springer Nature remains neutral with regard to jurisdictional claims in published maps and institutional affiliations.

## Figures and Tables

**Table 1:** Background characteristics of the study population.

Characteristic	Alginate (n = 50)	Antacid (n = 50)	p value
Maternal age (years)	29.0 ± 5.5	30.9 ± 5.4	0.095
Gestational age when heartburn present (weeks)	26.9 ± 8.0	23.6 ± 9.2	0.053
Pre-pregnancy BMI (kg/m^2^)	22.4 ± 4.7	21.9 ± 3.5	0.577
Multiparous	48 (96.0%)	48 (96.0%)	1.000
Twin pregnancy	1 (2.0%)	1 (2.0%)	1.000
History of heartburn before pregnancy	30 (60%)	23 (46%)	0.161
Frequency of heartburn (times per weeks)	13 (5, 20.2)	12 (7, 21)	0.649
Intensity of heartburn (VAS scores)	42.5 (31, 60)	43.5 (28.8, 60)	0.942

BMI: body mass index. VAS: visual analog scale. Data presented as mean ± standard deviation, median (interqurtile) or n (%).

**Table 2:** Heartburn after treatment, quality of life, maternal satisfaction, and maternal side effects.

	Alginate (n = 50)	Antacid (n = 50)	p value
Improvement of heartburn frequency	40 (80%).	44 (88%)	0.275
50% reduction of frequency in all-day of heartburn	28 (56%)	26 (52%)	0.688
Improvement of heartburn intensity	46 (92%)	46 (92%)	1.000
50% reduction of pain score of heartburn intensity	32 (68%)	40 (80%)	0.075
Quality of life			
SF-12v2 PCS (median change in score)	7.7 (0, 15.3)	7.6 (0, 15.9)	0.82
SF-12v2 MCS (median change in score)	11.4 (0.9, 21.7)	6.8 (0.5, 18.1)	0.352
Maternal Satisfaction			
Satisfied or very satisfied with treatment	40 (80%)	40 (80%)	1.000
Maternal side effect*			
Yes	24 (48%)	25 (50%)	0.841
Constipation	10 (20%)	13 (26%)	
Chalk-like taste	11 (22%)	7 (14%)	
Diarrhea	0	2 (4%)	
Bloating	7 (14%)	4 (8%)	
Nausea	4 (8%)	1 (2%)	
Paresthesia	2 (4%)	0	
Fatigue	1 (2%)	1 (2%)	

Data presented as median (interquartile) or n (%). *Some women had more than one side effect. VAS: Visual analog scale, PCS: physical health composite score, MCS: mental health composite score.

**Table 3:** Comparison of pregnancy and neonatal outcomes.

	Alginate (n = 50)	Antacid (n = 50)	p value
GA at delivery (weeks)	38.5 ± 1.3	38.8 ± 1.3	0.229
Mode of delivery			0.095
Vaginal	14 (28%)	22 (44%)	
Cesarean section	36 (72%)	28 (56%)	
Sex*			0.074
- Male	20 (39.2%)	29 (56.9%)	
- Female	31 (60.8%)	22 (43.1%)	
Birth weight (grams)*	3104.1 ± 455.8	3064.1 ± 395.2	0.637
APGAR score (at 1 minute) < 7*	2 (3.9%)	0 (0.0%)	0.495
APGAR score (at 5 minute) < 7*	1 (2.0%)	0 (0.0%)	1.000
NICU admission*	3 (5.9%)	1 (2.0%)	0.617

Data presented as mean ± standard deviation or n (%). GA: gestational age. *For neonatal outcomes: n = 51 per group.
